# Osteoclast development from peripheral blood monocytes is reduced in patients with radiographic axial spondyloarthritis on biological therapy

**DOI:** 10.1186/s13075-025-03578-9

**Published:** 2025-05-30

**Authors:** Cecilia Engdahl, Malin C. Erlandsson, Magnus Hallström, Anna Deminger, Helena Forsblad-d’Elia

**Affiliations:** 1 Department of Rheumatology and Inflammation Research, Institute of Medicine, Sahlgrenska Academy at University of Gothenburg, Göteborg, Sweden; 2 Department of Rheumatology, Sahlgrenska University Hospital, Region Västra Götaland, Göteborg, Sweden; 3 Sahlgrenska Osteoporosis Centre, Centre for Bone and Arthritis Research, Institute of Medicine, Sahlgrenska Academy, University of Gothenburg, Gothenburg, Sweden

**Keywords:** r-axSpA, Bone loss, Biological DMARDs, Osteoclast

## Abstract

**Background:**

Radiographic axial spondyloarthritis (r-axSpA) is a chronic inflammatory disease that primarily affects the axial skeleton and entheses, leading to pathological spinal bone formation and systemic bone loss. Treatments with tumor necrosis factor inhibitors (TNFi) and interleukin-17 inhibitors (IL-17i) have shown efficacy in reducing inflammation and potentially impacting bone remodeling in r-axSpA. Osteoclasts, crucial for bone resorption, are derived from the monocytic cell lineage and regulated by proinflammatory cytokines. This study aimed to evaluate the osteoclast development capacity from peripheral blood monocytes in patients with r-axSpA with different treatment strategies and compare it to controls.

**Methods:**

This study included 28 patients with long-standing r-axSpA receiving various treatments, including disease-modifying anti-rheumatic drugs (DMARDs) and NSAIDs, as well as 16 blood-donor controls. Disease activity was assessed using the Ankylosing Spondylitis Disease Activity Score (ASDAS). CD14 + monocytes were isolated from blood samples and differentiated into osteoclasts in vitro by stimulation with three different conditions: (I) macrophage colony-stimulating factor (M-CSF), (II) M-CSF and receptor activator of nuclear factor-κβ (RANKL), and (III) M-CSF, RANKL, and tumor necrosis factor-alpha (TNF). Osteoclast and osteoclast precursor formation were assessed using tartrate-resistant acid phosphatase (TRAP) staining, and TRAP5b concentration in supernatants was measured by ELISA.

**Results:**

The frequency of CD14 + monocytes was similar in patients with r-axSpA and controls, but the capacity to develop osteoclasts and osteoclast precursors was significantly decreased in the r-axSpA patients. Stratification of the patients based on treatment with or without biological DMARDs (bDMARDs) revealed no significant differences in ASDAS or frequency of CD14 + monocytes. Notably, only r-axSpA patients receiving bDMARDs exhibited a reduced ability to develop osteoclasts and osteoclast precursors compared to those not on bDMARDs and controls. Lower Trap5b concentrations in supernatants corroborated these findings.

**Conclusions:**

Our study demonstrates that patients with r-axSpA exhibit a reduced capacity for osteoclast formation from CD14 + monocytes isolated from peripheral blood. The process was modulated by treatment with bDMARDs, which might explain the previously shown sparing effect of bDMARDs on bone mineral density in r-axSpA.

**Supplementary Information:**

The online version contains supplementary material available at 10.1186/s13075-025-03578-9.

## Background

Radiographic axial spondyloarthritis (r-axSpA) or ankylosing spondylitis (AS), terms that are used interchangeably for the disease [1], is a chronic inflammatory disorder characterized by inflammation of the sacroiliac joints and spine. This inflammation impacts bone homeostasis, and we have previously shown that high C-reactive protein (CRP) is associated with reduced bone mineral density (BMD) in the femoral neck in patients with r-axSpA [2]. We have also shown in a national registry study that r-axSpA patients have a higher risk of both vertebral and non-vertebral fractures [3]. Further, r-axSpA is characterized by abnormal spinal new bone formation, which leads to the development of syndesmophytes and potentially results in total spinal ankylosis. Thus, r-axSpA is associated with pathological new bone formation in the spine and concurrent systemic bone loss at peripheral sites and within vertebrae.

The cells involved in bone remodeling come from distinct origins. Osteoblasts that synthesize new bone during skeletal development and remodeling originate from the mesenchymal cell line. In contrast, osteoclasts that break down bone arise from the hematopoietic cell line as monocytes, sharing a common lineage with macrophages and dendritic cells. All types of monocytes need macrophage-colony stimulating factor (M-CSF) for development and survival. Osteoclast differentiation is further regulated by the receptor activator of nuclear factor-κB ligand (RANKL), which binds to and activates its receptor RANK—a key signaling pathway essential for bone remodeling. Osteoblasts are the primary source of RANKL, but T-cells and other immune cells can also produce it. Single-nucleotide polymorphisms in the gene coding for RANKL (*RANKL)* are linked to syndesmophyte formation in r-axSpA [4], and we have previously shown that patients with r-axSpA have lower levels of soluble RANKL [5]. However, large meta-analyses indicate higher RANKL levels in r-axSpA. patients [6, 7]. These findings highlight the complexity of RANKL and bone regulation in r-axSpA.

Pro-inflammatory cytokines like tumor necrosis factors (TNF), interleukin (IL)-1, IL-6, and IL-17 can enhance RANKL expression, further promoting osteoclastogenesis. All cells of monocytic origin can differentiate into osteoclasts, but classical monocytes, which are the first responders to injury, are more prone to become osteoclasts [8, 9]. Despite high levels of pro-inflammatory cytokines, studies suggest that patients with r-axSpA do not have altered levels of classical monocytes in peripheral blood but have fewer non-classical monocytes involved in tissue resolving [9, 10]. Monocytes or circulating osteoclast precursors can be isolated from peripheral blood mononuclear cells (PBMC) using CD14 beads and then induced to become osteoclasts with RANKL and M-CSF. Osteoclasts can also be cultivated directly from all PBMC with extended RANKL and M-CSF stimulation. Previous osteoclast cultivation studies, initiated from PBMCs on patients with r-axSpA, indicate a reduced response to osteoclastogenic stimuli [8, 9].

The underlying pathogenic mechanisms of r-axSpA are not fully understood, but TNF and IL-17 are known to contribute to the disease mechanisms [11, 12]. Biological disease-modifying antirheumatic drugs (bDMARDs), such as TNF-inhibitors (TNFi) and IL-17 inhibitors (IL17i), reduce systemic inflammation, which in turn decreases osteoclast-mediated bone loss. In r-axSpA, TNFi treatment has been indicated to slow the spinal radiographic progression [13, 14] and maintain or improve BMD [14, 15]. The response to pro-osteoclastogenic stimuli and bone remodeling markers in serum were not affected by short-term treatment of TNFi in r-axSpA [16]. Still, patients with r-axSpA had lower levels of osteoclasts at baseline compared to controls. Treatment with IL-17i for two years improved symptoms and the spinal radiographic progression was low [17]. Like TNFi, IL-17i maintained BMD and did not alter the bone turnover biomarkers in patients with r-axSpA [18]. In addition, both TNFi and especially IL17i promote osteoblast function by reducing Dickkopf-1, a protein that inhibits the WNT pathways crucial for osteoblast differentiation [9, 19, 20, 21].

This study aims to test the hypothesis that osteoclast precursors in the blood of patients with long-standing r-axSpA have a reduced ability to respond to osteoclastogenic stimuli compared to controls and that treatment with bDMARD influences this response.

## Methods

### Patients

The patients were recruited from a cohort of patients with long-standing r-axSpA in western Sweden who participated in a longitudinal study that started in 2009. The inclusion in 2009 has been described previously [22]. Inclusion criteria were AS according to the modified New York criteria [23] and age ≥ 18 years. Exclusion criteria were psoriasis, inflammatory bowel disease, ongoing pregnancy, dementia, or difficulties understanding the Swedish language. All patients included in 2009 were invited to a 13-year follow-up study in 2022–2023, the Long-term Outcome Ankylosing Spondylitis (LOAS) study. The present analyses included a subset of the patients participating in the 13-year follow-up, assessed between the 12th of January 2022 and the 20th of December 2022.

Patients were assessed using questionnaires that included medical history and medications, including non-steroid anti-inflammatory drugs (NSAID) and conventional and biological disease-modifying anti-rheumatic drugs (DMARDs). Disease activity was assessed using the AS disease activity score (ASDAS) based on C-reactive protein (CRP) [24]. High-sensitivity CRP (hs-CRP), Erythrocyte Sedimentation Rate (ESR), Hemoglobin (Hb), White Blood Cell Count (WBC), Platelet Count (PLT), Alanine Aminotransferase (ALT), and creatinine were analyzed using standard laboratory techniques.

#### Controls

The control group for the r-axSpA 13-year follow-up, LOAS, was collected at the local blood donor centre in Gothenburg. A subset of these patients, collected between December 7th, 2022, and March 23rd, 2023, was used as the controls for this specific study.

#### Sample collection

PBMCs were collected from the cubital vein using heparinized BD Vacutainer^®^ CPT™ Mononuclear Cell Preparation Tubes (Becton Dickinson, Franklin Lakes, New Jersey, USA) according to the manufacturer’s instructions. Cells were counted using an automated cell counter (Sysmex, Europe GmBH, Norderstedt, Germany) and stored − 80 °C in bovine fetal serum (Sigma Aldrich, Saint Louis, Missouri, USA) supplemented with 10% DMSO (Sigma Aldrich).

#### Osteoclast differentiation

PBMCs were thawed and counted by automated cell counter, and CD14 + cells were sorted using positive selection with magnetic beads on MACS LS separation column according to the manufacturer’s instructions (Milteny Biotec, Cologne, Germany). The resulting CD14 + cells were calculated with a nucleocounter SP-100 (Chemometec, Allerod, Denmark), and the frequency was calculated from all thawed PBMCs.

CD14 + cells were seeded 80.000 per well in 384 well cell culture plates (Corning, Corning, New York, USA) in alpha-MEM (Gibco, Thermo Fisher, Waltham Massachusetts, USA) supplemented with 10% fetal bovine serum (Sigma-Aldrich), 1% Glutamax (Gibco) and 0.1% penicillin-streptomycin (Gibco, Thermo Fisher). Cells were stimulated with macrophage-colony stimulating factor (M-CSF), 30 ng/ml (RnD systems, Minneapolis, MN, USA), with or without the addition of receptor activator of nuclear factor-κβ (RANKL), 4ng/ml (RnD systems) and tumor necrosis factor-alpha (TNF), 2.5 ng/ml (RnDsystems).

Cells were incubated in a standard 37 °C incubator under a 5% CO2 atmosphere for 3 + 2 days with an exchange of media after three days.

#### Cell staining and documentation

At harvest, cells were fixed with 72% acetone in citrate buffer and stained for tartrate-resistant alkaline phosphatase (TRAP) activity using the acid phosphatases leukocyte kit according to the manufacturer’s instructions (Sigma-Aldrich). The stained plates were dried, and each well was photographed with a digital microscope (Nikon, Tokyo, Japan). The images were examined using the software ImageJ (NIH, USA). Pre-osteoclasts and osteoclasts were quantified, TRAP-positive multinucleated (with two nuclei) cells were defined as pre-osteoclasts, and TRAP-positive multinucleated (with more than three nuclei) cells were defined as osteoclasts. Samples from 69 individuals (52 patients, 17 controls) were stimulated for osteoclast differentiation. Some samples were excluded from all analyses due to low cell viability in cultivation (24 patients and one control). Including samples with non-countable cells in the M-CSF stimulated wells (19 patients) or no TRAP-positive cells in M-CSF and RANKL stimulated wells (5 patients and one control).

#### Quantification in supernatants

At harvest and media exchange, supernatants were collected and kept at -20 °C. The supernatants at harvest were analyzed for TRAP5B activity using the BoneTRAP^®^ (TRAP 5b) ELISA (ImmunoDiagnostic Systems, Tyne & Wear, United Kingdom), and Interleukin 6 levels were measured in supernatants from day 3 (medium exchange) using the Pelikine compact IL6 ELISA kit (Sanquin, Amsterdam, The Netherlands).

#### Statistics

Descriptive statistics are presented as medians with 25th to 75th percentile and frequencies as numbers with percentages. Statistical analyses were performed using GraphPad Prism version 10 (GraphPad Software, La Jolla, San Diego, CA, USA). Mann-Whitney U tests were applied for comparisons between two groups (controls and r-axSpA patients), while Kruskal-Wallis tests followed by Dunn’s multiple comparisons were used for analyses involving multiple groups (controls, r-axSpA patients with and without bDMARDs, and patients categorized by different bDMARDs). A *p*-value < 0.05 was considered statistically significant.

#### Ethics approval

This study’s procedures were performed in accordance with the Declaration of Helsinki. The Swedish Ethical Review Authority approved the study for patients and controls (registration 2021–03484).

## Results

### Patients and controls

Characteristics of 28 patients with long-standing r-axSpA and 16 controls are shown in Table 1. The patients with r-axSpA were 64 years (median), and the controls were 57 years (median), with no significant statistical difference. The sex distribution in patients and controls was similar; 61% versus 62% were males in the different groups. The symptom duration was a median of 33 years, and 82% of the patients were HLA-B27 positive. A proportion of the patients were treated with NSAIDs as well as conventional DMARDs and/or bDMARDs.

**Table 1:** Characteristics of patients with radiographic axial spondyloarthritis and controls

	Controls *n* = 16	r-axSpA *n* = 28	*P* value
Age (year)	57 [46–66]	64 [54–71]	0.07
Male number and age (year) Female number and age (year)	10 (62%) 48 [45–64] 6 (38%) 61 [56–66]	17 (61%) 61 [51–71] 11 (39%) 64 [52–73]	1.0 0.06 0.61
Symptom duration (year)		33 [25–44]	
HLA-B27+		23 (82%)	
NSAID		8 (29%)	
cDMARDS		4 (15%)	
bDMARDS			
TNFi		10 (36%)	
IL-17i		3 (11%)	
ASDAS, score		1.90 [1.34–2.65]	
CRP (mg/L)		2.30 [2.00-4.50]	
ESR (mm/*h*		11.0 [6.0-22.5]	
Hb (g/L)		145 [141–150]	
WBC (10^9^/L)		5.9 [5.4–7.3]	
PLT (10^9^/L)		249 [236–291]	
ALT (*µkat*/L)		0.41 [0.34–0.52]	
Creatinine (*µmol*/L)		77 [73–87]	

Values are stated as medians with 25th to 75th percentile or numbers with percentages. The Mann-Whitney U-test was used to compare the control group and patients with radiographic axial spondyloarthritis. r-axSpA; radiographic axial spondylarthritis, HLA-B27; Human leukocyte antigen B27, NSAID; non-steroidal anti-inflammatory drug, cDMARDS; conventional disease-modifying antirheumatic drugs, bDMARDS; biological disease-modifying antirheumatic drugs, TNFi; tumour necrosis factor inhibitor, IL17i; Interleukin-17 inhibitor, ASDAS; Ankylosing Spondylitis Disease Activity Score, CRP; C-reactive protein. ESR; Erythrocyte Sedimentation Rate, Hb: Hemoglobin, WBC; White Blood Cell Count, PLT; Platelet Count, ALT; Alanine Aminotransferase

### Osteoclast precursors and osteoclasts in patients with r-axSpA and controls

**Fig. 1:**
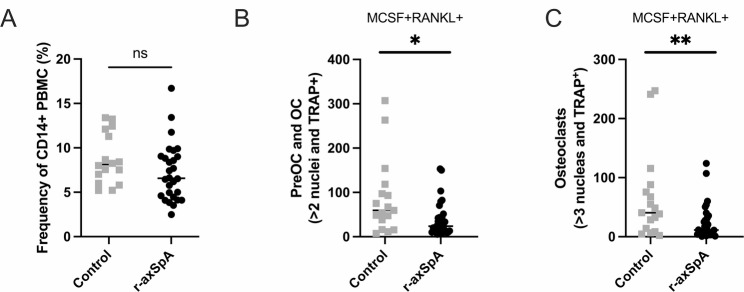
Monocyte and osteoclast differentiation in patients with radiographic axial spondylarthritis (r-axSpA) and controls: (**a**) Frequency of CD14+ (monocytes) in peripheral blood mononuclear cells (PBMCs). (**b**) The number of osteoclasts precursors (preOCs) and osteoclasts (OCs), defined as more than two nuclei and tartrate-resistant alkaline phosphatase (TRAP) positive in a 384-well plate. (**c**) The number of osteoclasts, defined as more than three nuclei and TRAP-positive in a 384-well plate. Statistical evaluations were performed using the Mann-Whitney test. All data are presented in a scatter dot plot. *p < 0.05, **p < 0.01

Performing CD14 purification showed a similar frequency of monocytes in controls 8.1% [6.8–11.5] and r-axSpA patients 6.8% [4.6-9.0], Fig. 1A. As a negative control, samples were plated with only M-CSF, which stimulated the survival of monocytes but not the development of osteoclasts. The M-CSF-stimulated cell populations displayed surviving monocytes but no osteoclasts or pre-osteoclasts (TRAP-positive and multinucleated cells) (data not shown). When RANKL and M-CSF were added to the cultivations, pre-osteoclasts and osteoclasts developed. The number of pre-osteoclasts and osteoclasts were lower in the r-axSpA patients, with a median of 25 [17–47] (pre-osteoclast and osteoclast per well) compared to controls median of 63 [45–102] (pre-osteoclast and osteoclast per well), Fig. 1B-C.

### Osteoclast precursors and osteoclasts in r-axSpA patients with and without bDMARDs

Patients with r-axSpA were further stratified into those with or without bDMARDs, as shown in Table 2.

**Table 2:** Characteristics of patients with radiographic axial spondylarthritis with and without bDMARDs

	*r*-axSpA, without-bDMARDs, *n* = 15	r-axSpA, with bDMARDs, *n* = 13	*P* value
Age (year)	64 [59–73]	64 [51–68]	*p* = 0.17
Male number and age (year) Female number and age (year)	10 (67%) 61 [59–65] 5 (33%) 71 [64–73]	7 (54%) 55 [50–66] 6 (46%) 64 [53–70]	*p* = 0.71 *p* = 0.98 *p* = 0.40
Height (cm)	174 [169–180]	173 [168–179]	*p* = 0.70
Weight (kg)	86.5 [71.5–92.8]	80.0 [71.0-85.2]	*p* = 0.39
Symptom duration (year)	33 [25–44]	29 [27–37]	*p* = 0.56
HLA-B27+	12 (80%)	11 (85%)	*p* = 1.0
NSAID	5 (33%)	3 (23%)	*p* = 1.0
cDMARDS	0 (0%)	4 (31%)	*p* = 0.02
ASDAS, score	2.7 [1.52–2.64]	1.68 [1.20–2.62]	*p* = 0.50
CRP (mg/L)	2.00 [2.00-5.80]	2.60 [2.00-3.50]	*p* = 0.88
ESR (mm/*h*	12.5 [5.3–17.0]	10.0 [7.0–30.0]	*p* = 0.64
Hb (g/L)	148 [142–158]	145 [138–146]	*p* = 0.08
WBC (10^9^/L)	6.3 [5.6–8.3]	5.6 [5.4–5.9]	*p* = 0.07
PLT (10^9^/L)	246 [232–267]	250 [238–304]	*p* = 0.37
ALT (*µkat*/L)	0.41 [0.33–0.54]	0.41 [0.36–0.48]	*p* = 0.95
Creatinine (*µmol*/L)	85 [76–98]	75 [68–78]	*p* = 0.18

Values are stated as medians with 25th to 75th percentile or numbers with percentages. The Mann-Whitney U-test was used to compare patients with radiographic axial spondyloarthritis with and without biological disease-modifying antirheumatic drugs. r-axSpA; radiographic axial spondylarthritis, bDMARDS; biological disease-modifying antirheumatic drugs, HLA-B27; Human leukocyte antigen B27, NSAID; non-steroidal anti-inflammatory drug, cDMARDS; conventional disease-modifying antirheumatic drugs, ASDAS; Ankylosing Spondylitis Disease Activity Score, CRP; C-reactive protein, ESR; Erythrocyte Sedimentation Rate, Hb: Hemoglobin, WBC; White Blood Cell Count, PLT; Platelet Count, ALT; Alanine Aminotransferase

**Fig. 2:**
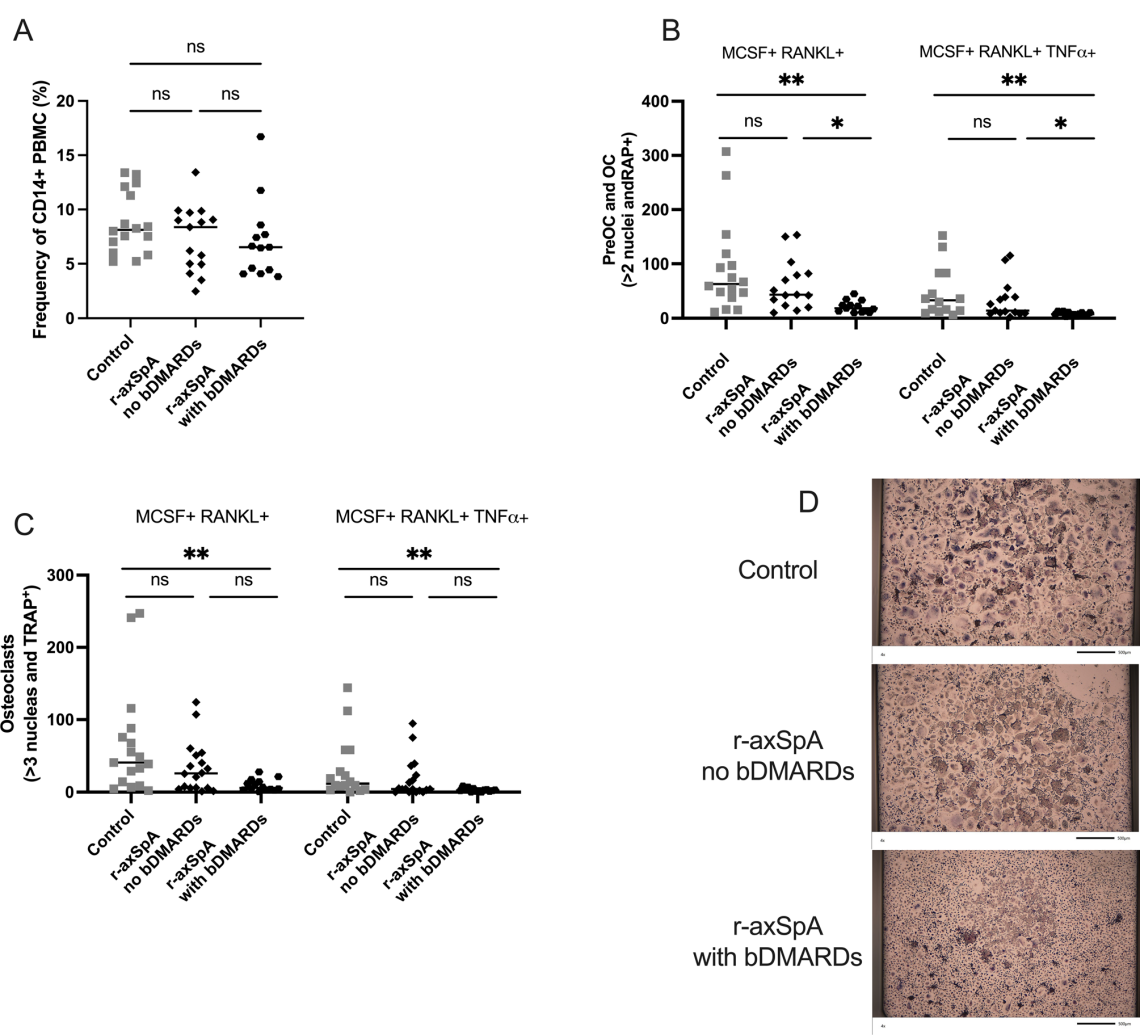
Difference in monocyte and osteoclast differentiation depending on biological DMARDs (bDMARDs) in patients with radiographic axial spondylarthritis (r-axSpA) and controls: (**a**) Frequency of CD14+ (monocytes) in peripheral blood mononuclear cells (PBMCs). (**b**) The number of osteoclasts precursors (preOCs) and osteoclasts (OCs), defined as more than two nuclei and tartrate-resistant alkaline phosphatase (TRAP) positive in a 384-well plate. (**c**) The number of osteoclasts, defined as more than three nuclei and TRAP-positive in a 384-well plate. (**d**) Representative pictures from the cultivation stimulated with M-CSF and RANKL. Statistical evaluations were performed using the Kruskal-Wallis followed by Dunns multiple comparisons of all groups compared to each other. All data are presented in a scatter dot plot. **p < 0.01

There were no differences in age, gender, symptom duration, occurrence of HLA-B27 positivity, use of NSAIDs, ASDAS, or various laboratory measurements between the groups. However, none of the patients without bDMARDs was treated with conventional DMARDs. The frequency of isolated CD14 + monocytes from PBMC was not altered, r-axSpA patients with bDMARD had 6.7% [4.5–7.6] and those without bDMARD had 8.4% [5.0-9.2] compared to controls 8.1% [6.8–11.5] (Fig. 2A). When investigating osteoclast development potential, the r-axSpA patients on bDMARDs showed a median of 22 [17–25] (osteoclast per well), compared to r-axSpA patients without bDMARDs who showed a median of 43 [22–80] (osteoclast per well), and controls of 63 [45–102] (osteoclast per well) (Fig. 2B and D). Additionally, the number of osteoclasts was only significantly altered in r-axSpA patients on bDMARDs with a median of 11 [5–16] (osteoclast per well), compared to controls 44 [25–75] (osteoclast per well). The r-axSpA patients without bDMARDs had a median of 26 [7–44] (osteoclast per well), (Fig. 2C-D). Regarding bDMARDs, most patients used TNFi, and a smaller proportion used IL17i (3/13, 23%). We separated these treatments, showing a significant reduction in pre-OC and osteoclast in TNFi-treated patients compared to controls and a clear tendency comparing IL17i-treated patients and controls (Supplementary Fig. 1). There was only a tendency between no-bDMARDs and TNFi-treated patients. Finally, we also investigated the additive effects of adding TNF to the cultivation. Adding TNF mimicked the results of RANKL stimulation alone, with reduced levels of preosteoclasts and osteoclasts in patients treated with bDMARS (Fig. 2B-C).

**The TRAP5b and IL6 concentration in supernatants from cultures from r-axSpA patients with and without bDMARDs**.

**Fig. 3:**
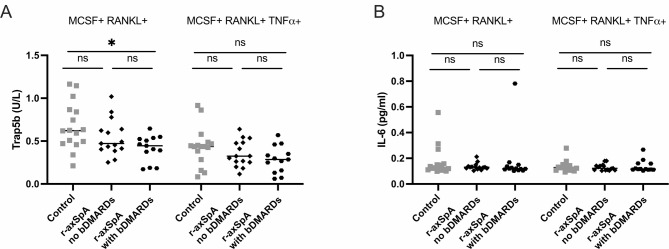
Differences in the supernatant of osteoclast cultivation in patients with radiographic axial spondylarthritis (r-axSpA) with the use of biological DMARDs (bDMARDs) and controls: (**a**) Tartrate-resistant alkaline phosphatase (TRAP) TRAP5b measured in the supernatant just before the harvest of osteoclast cultivation. (**b**) IL-6 was measured in the supernatant on the third day of cultivation before most cells became osteoclast. Statistical evaluations were performed using the Kruskal-Wallis followed by Dunns multiple comparisons of all groups compared to each other. All data are presented in a scatter dot plot. *p < 0.05

The concentration of TRAP5b, a marker specific for osteoclast development, was measured in the cell-culture supernatants of M-CSF and RANKL-stimulated cells at harvest, i.e., after 3 + 2 days of culture. TRAP5b was reduced in r-axSpA patients on bDMARDs compared to controls. These differences disappeared after stimulation with TNF (Fig. 3A). No differences were found in IL-6 concentration (Fig. 3B).

## Discussion

This study demonstrates that peripheral blood monocytes from patients with long-standing r-axSpA exhibit a reduced ability, compared to controls, to differentiate into osteoclasts, primarily influenced by treatment with bDMARDs.

Our cohort consisted of patients with well-established r-axSpA with a symptom duration of a median of 33 years and with different treatments. It is crucial to note that these findings may not apply to newly diagnosed patients.

We found no differences in the frequency of circulating classical monocytes (CD14-positive cells) in r-axSpA patients compared to controls. This is in line with the previous study of Perpétuo et al., who found no differences in classical or intermediate monocytes but noted a decreased frequency of non-classical monocytes (CD14^dim^CD16^+^) [9]. Surdacki et al. also reported a lower frequency of non-classical monocytes and, in contrast to our results, a higher frequency of classical monocytes in r-axSpA patients compared to controls [25]. In addition, Huang et al. reported a higher monocyte-to-lymphocyte ratio in well-established patients with r-axSpA than controls [26]. While classical monocytes are the primary precursors for osteoclasts, all monocytes can differentiate into osteoclasts under appropriate stimuli.

We assessed the osteoclastogenic ability by stimulating monocytic lineage cells from blood samples with RANKL and M-CSF. In autoimmune diseases, the altered blood milieu, influenced by pro-inflammatory cytokines, affects blood cells, including monocytes. Korkosz et al. demonstrated that sera from r-axSpA patients exhibited lower levels of RANKL, yet further stimulated the development of osteoclasts from unrelated monocytes and induced RANKL stimulation in culture compared to sera from healthy controls [27]. This suggests that the sera from r-axSpA have a stimulatory effect independent of RANKL, which promotes osteoclast formation.

This indicates that CD14-positive circulating monocytes from r-axSpA patients are less prone to osteoclast differentiation and may have a more straightforward response to immune-activated. Previous studies also reported less osteoclast development from all PBMC in patients with r-axSpA when stimulated with RANKL [9, 10]. The difference in osteoclast cultivation from all peripheral blood mononuclear cells contra purified monocytes might reveal different interesting aspects. Purified monocytes might better show disease- and treatment-related alterations of the monocytes by excluding other cell types that can influence differentiation.

Although we consistently aimed to stimulate the same number of cells, cells were uncountable in 25 samples in the M-CSF-stimulated group or M-CSF and RANKL-stimulated osteoclast. This is likely attributable to sample variability, with some requiring more extensive or prolonged stimulation and may also be related to the storage duration. The cells from non-responders in r-axSpA were stored for a longer period compared to those from responders, while the cells from the controls were stored for a shorter period.

The direct relationship between bDMARDs and osteoclastogenesis needs to be further investigated. bDMARDs reduce disease activity, and studies have shown an association between TNFi and IL-17i and limited bone loss [14, 15, 16, 17, 18, 28, 29, 30, 31, 32]. TNF and IL-17 are also known to stimulate RANKL-induced osteoclastogenesis directly [33, 34, 35]. Perpétuo et al. conducted studies on osteoclasts in patients with rheumatoid arthritis (RA) and r-axSpA before and after TNF inhibitor (TNFi) treatment [16, 35]. In RA, TNFi-treatment decreased the frequency of circulating classical monocytes and the number of osteoclasts developed from total peripheral blood mononuclear cell (PBMC) stimulation with RANKL and M-CSF [35]. In r-axSpA patients, TNFi treatment showed no changes in the frequency of monocytes or PBMC-stimulated osteoclasts compared to baseline; however, a higher resorption capacity was measured in the osteoclast cultivation on bone surface [16]. In addition, r-axSpA patients exhibited a lower frequency of monocytes and osteoclast numbers compared to the controls. Our study, comparing patients with and without bDMARDs, revealed a significant decrease in osteoclastogenic potential in those on bDMARDs. This is to our knowledge the first study to show that the use of bDMARDs is associated with lower osteoclastogenic potential from circulating monocytes in r-axSpA patients.

In addition to RANKL stimuli, we also investigated the additive effect of TNF on osteoclastogenesis. In murine models, TNF has a stimulatory effect during continued cultivation of osteoclasts [34]. In human osteoclastogenesis, the effect is more debated, in vivo TNF can stimulate surrounding cells to higher expression of RANKL. We were mainly interested in the impact of TNF stimulation in bDMARDs-treated r-axSpA patients. However, our results showed no additional effects of TNF with RANKL compared to RANKL alone. This may be attributed to our method with continuous TNF stimulation during cultivation, which could keep monocytes in an inflammatory state, inducing them to become activated macrophages instead of osteoclasts [36].

Finally, we investigated the protein secreted in the supernatant of the osteoclast cultivations. We found that Trap5b, an osteoclast marker, decreased in the supernatant from bDMARD-treated r-axSpA patients. This aligns with our results, which showed lower numbers of pre-osteoclasts and osteoclasts and is consistent with a previous publication on osteoclast cultivation [37]. There was no effect on IL-6 secretion, indicating no alteration of the inflammatory status in cell cultivation between CD14 + cells from patients with r-axSpA and controls.

There are limitations in this study. Due to technical issues, we could not process 25 samples due to low cell viability during cultivation, likely resulting from decreased survival during more extended storage. The non-responder cells were stored in 337 [169–268] days, while the responders were stored in 138 [57–234] days (data not shown). Consequently, the study population was restricted to only 16 controls and 28 individuals with r-axSpA. Additionally, there was a wide age span and a tendency for a higher age in the r-axSpA patients compared to the controls, explained by a trend of higher age in the male patients. Nonetheless, we could still demonstrate a difference in osteoclast development between the r-axSpA and control group and between patients with and without bDMARDs. The study’s cross-sectional nature prevents us from evaluating the effect of initiating treatment with bDMARDs on osteoclast development. Furthermore, we did not consider the duration of the treatment with bDMARDs.

## Conclusions

To our knowledge, this is the first study to demonstrate a direct connection between bDMARD use and the osteoclastogenic potential of blood monocytes in r-axSpA patients. The observed protective effect on bone mass associated with bDMARDs in patients with r-axSpA may be linked to the reduction of osteoclastogenic capacity.

## Electronic supplementary material

Below is the link to the electronic supplementary material.

**Supplementary Material 1**: **Supplementary Figure 1**: Differences in osteoclast differentiation depending on types of biological DMARDs (bDMARDs), Interleukin-17 Inhibition (IL17i), or tumor necrosis factor-alpha Inhibition (TNFi) in patients with radiographic axial spondylarthritis (r-axSpA) and controls.(a) The number of osteoclast precursors (preOCs) and osteoclasts (OCs), defined as more than two nuclei and tartrate-resistant alkaline phosphatase (TRAP) positive in a 384-well plate. (b) The number of osteoclasts, defined as more than three nuclei and TRAP-positive in a 384-well plate. Statistical evaluations were performed using the Kruskal-Wallis followed by Dunns multiple comparisons of all groups compared to each other. All data are presented in a scatter dot plot. *p < 0.05.

## Data Availability

The data sets generated and/or analyzed during the current study are not publicly available due to the General Data Protection Regulation (GDPR), but a limited data set that supports the main analyses is available on reasonable request.

## Abbretionsvia

r-axSpA: Radiographic axial spondyloarthritis
TNFi: Tumor necrosis factor inhibitors
IL-17i: Interleukin-17 inhibitors
DMARDs: Disease-modifying anti-rheumatic drugs
ASDAS: Ankylosing spondylitis disease activity score
NSAID: Non-steroid anti-inflammatory drugs
M-CSF: Macrophage colony-stimulating factor
RANKL: Receptor activator of nuclear factor-κβ
TNF: Tumor necrosis factor-alpha
TRAP: Tartrate-resistant acid phosphatase
bDMARDs: Biological DMARDs
AS: Ankylosing spondylitis
CRP: C-reactive protein
BMD: Bone mineral density
RANK: Receptor activator of nuclear factor-κβ
IL: Interleukin
PBMC: Peripheral blood mononuclear cells
RA: Rheumatoid arthritis
ESR: Erythrocyte Sedimentation Rate
Hb: Hemoglobin
WBC: White Blood Cell Count
PLT: Platelet Count
ALT: Alanine Aminotransferase
