# Supplementary figures and images for: Osteoclast development from peripheral blood monocytes is reduced in patients with radiographic axial spondyloarthritis on biological therapy
Source: Arthritis Res Ther. 2025 May 30;27:117. doi: 10.1186/s13075-025-03578-9 (PMC12123737; doi:10.1186/s13075-025-03578-9)

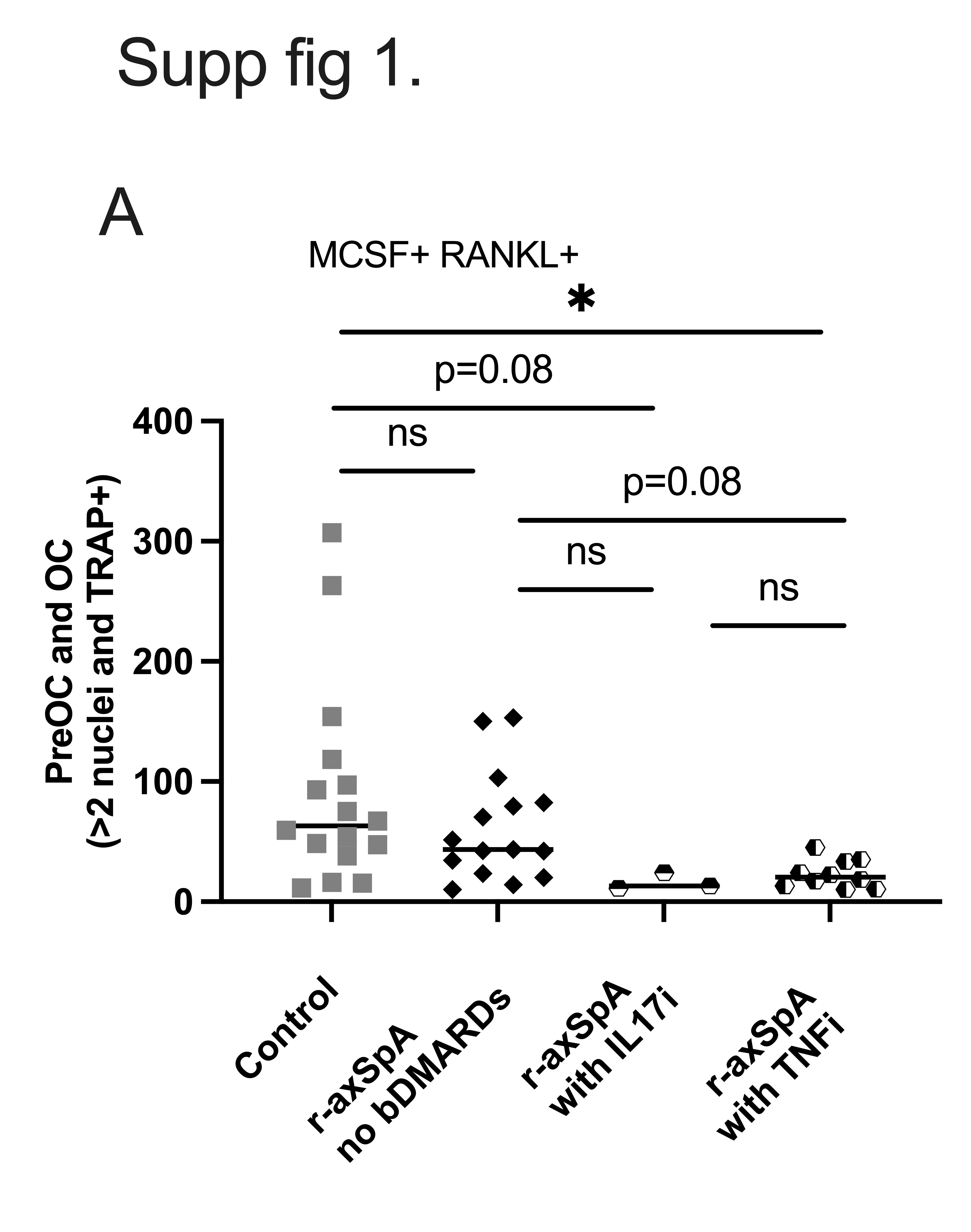

Supplement: Supplementary file 1 — Supplementary Material 1: Supplementary Figure 1: Differences in osteoclast differentiation depending on types of biological DMARDs (bDMARDs), Interleukin-17 Inhibition (IL17i), or tumor necrosis factor-alpha Inhibition (TNFi) in patients with radiographic axial spondylarthritis (r-axSpA) and controls.(a) The number of osteoclast precursors (preOCs) and osteoclasts (OCs), defined as more than two nuclei and tartrate-resistant alkaline phosphatase (TRAP) positive in a 384-well plate. (b) The number of osteoclasts, defined as more than three nuclei and TRAP-positive in a 384-well plate. Statistical evaluations were performed using the Kruskal-Wallis followed by Dunns multiple comparisons of all groups compared to each other. All data are presented in a scatter dot plot. *p < 0.05. [file 13075_2025_3578_MOESM1_ESM.tiff]
